# Supplementary material for: Risk Prediction Models for Melanoma: A Systematic Review on the Heterogeneity in Model Development and Validation
Source: Int J Environ Res Public Health. 2020 Oct 28;17(21):7919. doi: 10.3390/ijerph17217919 (PMC7662952; doi:10.3390/ijerph17217919)
Supplement: Supplementary file 1 [file ijerph-17-07919-s001.pdf]

Supplementary File 1: Complete search strategy

The search string for the literature search in Pubmed was:

((melanoma*[tiab] OR 'skin cancer'[tiab]) AND ('risk factor'[tiab] OR 'risk assessment'[tiab] OR 'probability'[tiab] OR 'likelihood'[tiab] OR chance[tiab] OR risk*[tiab]) AND ('prediction'[tiab] OR 'model'[tiab] OR 'score'[tiab] OR predict*[tiab])) AND (("2013/05/01"[Date - Publication] : "3000"[Date - Publication]))

Supplementary Table S1. Classification of model variables into meaningful groups

Original variables	References	New variable(s) used for analysis
Skin color	[16,22,23,29,34,35,54,56]	Skin color
Melanin density	[19]	
Colorimetric variables	[17]	
Freckles in childhood	[16,45]	Freckles
Freckling	[22,51,56]	
Freckles	[23,31]	
Freckle density	[32,37]	
Freckling tendency	[30]	
Density of freckles on arms before age 20	[36]	
Freckling as an adult	[62]	
Sunburns in all life	[16,22,30,51,56]	Sunburns
Sunburn	[18,34,46]	
Sunburns in childhood	[23,36,37,45,62]	
Sun exposure > 240 hours/year	[21]	Sun exposure
Mean time spent outdoors in summer aged 10-24	[20]	
Sunbathing vacations	[33]	
Intermitted sun exposure	[57]	
Vacational sun exposure	[62]	
Total childhood sun exposure hours	[45]	
Outdoor UV	[39]	
Nevi	[18,28,33,37,45,50,62]	Nevi
Raised nevi	[20]	
Melanocytic nevi	[21,35]	
Moles	[22,61]	
Common nevi	[23,31,46,53,57,60]	
Melanocytic common nevi	[24,25]	
Benign nevi	[30]	
Nevi density	[32,49,56]	
Raised moles	[36]	
Nevi distribution	[51]	
Moles at age 21	[52]	
Keratinocytic cancer	[50]	Non-melanoma skin cancer
Past history of excisions for skin cancer (not melanoma)	[52]	
Personal history of non-melanoma skin cancer	[20,31,36,45,62]	
Actinic lentigines	[24,25]	Sun damage
Solar lentigines	[45,50]	
Solar skin damage	[22,57]	
UV damage	[28]	

**Original variables**	**References**	**New variable(s) used for analysis**
Atypical nevi	[21,24,25,30,31,53]	Atypical nevi
Dysplastic nevi	[29,57]	
Tanning ability	[22,29,34,45,52,54,59]	Tanning ability
Skin reaction to repeated sun exposure	[32]	
Regular dermatologist visits	[26]	Skin checks
Skin checks in the past 3 years	[52]	
Indoor tanning	[56]	Sunbed sessions
Sunbed use	[33,57]	
Sunbank sessions	[45,49,62]	
Indoor UV	[39]	
Place of residence	[37]	Residence
Country	[60]	
Life in a country at low latitude	[35]	
Red hair (RH)-phenotype	[46]	Hair color
		Freckles
		Fitzpatrick
Pigmentation score	[45]	Tanning ability
		Fitzpatrick,
		Skin color
		Eye color
		Hair color
		Freckles

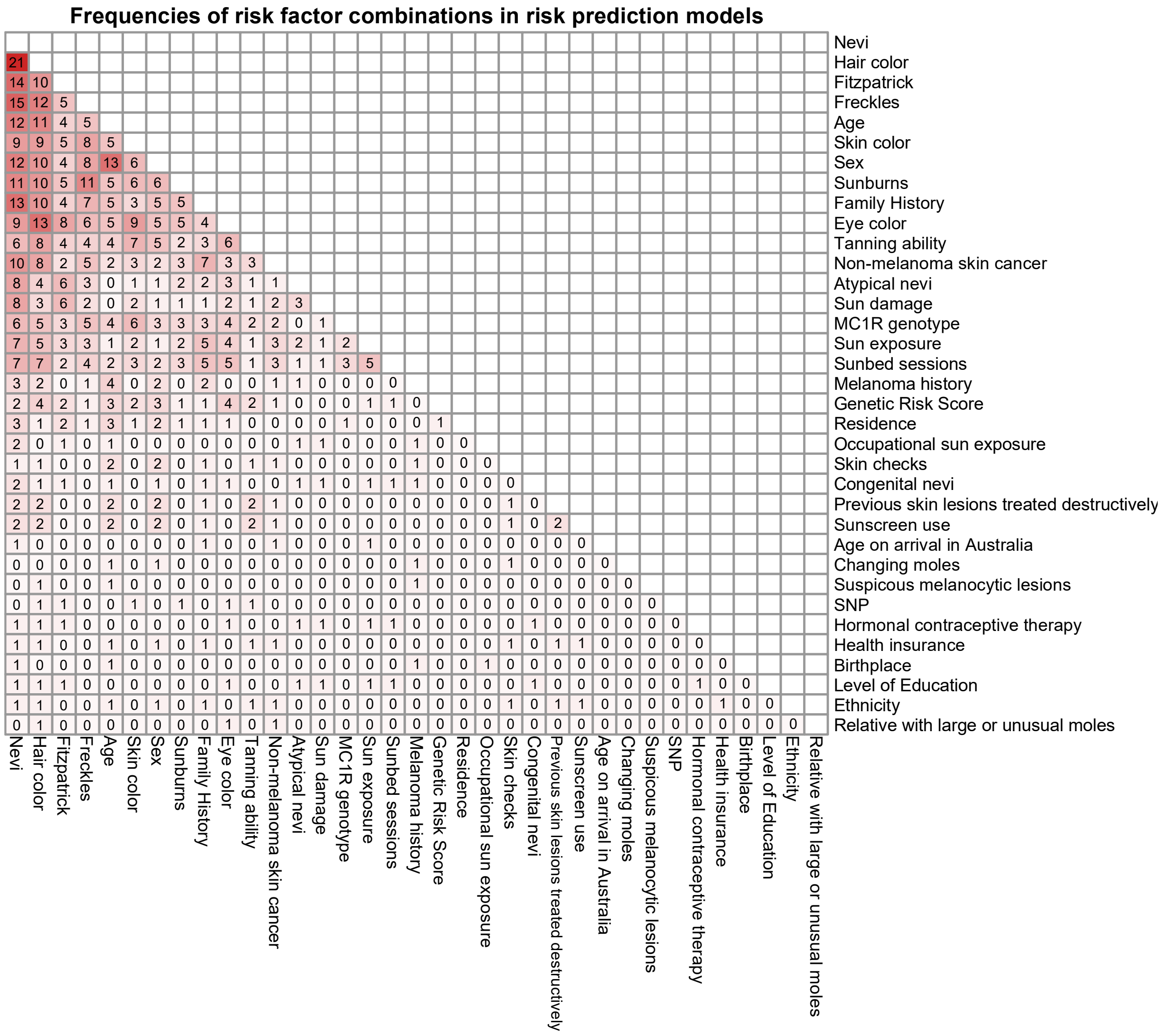

Supplementary Figure S1. Heatmap indicating relations joint occurrences of risk factor pairs among in risk prediction models for melanoma. All 35 risk factors occurring in any of the risk prediction models are included. Each number represents the absolute frequency of the corresponding risk factor combination. The darker the field the more frequent is the corresponding risk factor combination (N = 45 models). MC1R = melanocortin 1 receptor, SNP = Single Nucleotide Polymorphism

Supplementary Table S2. Absolute (n) and relative (%) frequencies of methods used when evaluating risk prediction models for melanoma regarding the methodological type of validation and the type of measures describing model performance (N=40 studies). With references (ref.).

	**Studies published up to 2011 (n=20)**			**Studies published after 2011 (n=20)**			**All Studies (N=40)**		
**Validation**	**n**	**%**	**ref.**	**n**	**%**	**ref.**	**n**	**%**	**ref.**
**Internal validation**	**5**	**25.0**	**[17,18,20,36,37]**	**13**	**65.0**	**[27,34,45,49, 50,52,54-57, 60-62]**	**18**	**45.0**	**[17,18,20,27,34, 36,37,45,49,50,52, 54-57,60-62]**
Cross-validation	1	5.0	[17]	5	25.0	[34,54,57,60, 62]	6	15.0	[17,34,54,57,60, 62]
Split sample	3	15.0	[18,20,36]	3	15.0	[52,55,56]	6	15.0	[18,20,36,52,55, 56]
Bootstrapping	1	5.0	[37]	5	25.0	[27,45,49,50, 61]	6	15.0	[27,37,45,49,50, 61]
**External validation**	**1**	**5.0**	**[23]**	**5**	**25.0**	**[49-51,59,62]**	**6**	**15.0**	**[23,49,50,51,59, 62]**
Both internal and external validation	0	0.0		3	15.0	[49,50,62]	3	7.5	[49,50,62]
Neither internal nor external validation	14	70.0	[19,21,22,24- 26,28-33,35, 40]	5	25.0	[16,39,46,53, 58]	19	47.5	[16,19,21,22,24- 26,28-33,35,39, 40,46,53,58]
**Performance measures**	**n**	**%**	**ref.**	**n**	**%**	**ref.**	**n**	**%**	**ref.**
**Calibration 1)**	**2**	**10.0**	**[18,37]**	**9**	**45.0**	**[45,49,50,52- 54,56,57,61,6 2]**	**11**	**27.5**	**[18,37,45,49,50,52 -54,56,57, 61,62]**
Hosmer-Lemeshow test	2	10.0	[18,37]	7	35.0	[45,50,54,56, 57 ,61,62]	9	22.5	[18,37,45,50,54,56 ,57,61,62]
Graph (plot/intercept/slope)	0	0.0		3	15.0	[49,52,61]	3	7.5	[49,52,61]
Calibration in the large	0	0.0		1	5.0	[49]	1	2.5	[49]
**Discrimination 2)**	**9**	**45.0**	**[17-20,23,28, 35-37]**	**20**	**100.0**	**[16,27,34,39, 45,46,49, 50-62]**	**29**	**72.5**	**[16-20,23,27,28, 34-37,39,45,46, 49,50-62]**
AUC	8	40.0	[17-19,23,28, 35-37]	18	90.0	[16,27,34,39, 45,46,49-51, 54-62]	26	65.0	[16-19,23,27,28, 34-37,39,45,46, 49,50,51,54-62]
C-index	0	0.0		3	15.0	[52,53,58]	3	7.5	[52,53,58]
Discrimination slope	0	0.0		1	5.0	[45]	1	2.5	[45]
ROC plot (without AUC calculation)	1	5.0	[20]	0	0.0		1	2.5	[20]
**Overall model performance 3)**	**0**	**0.0**		**1**	**5.0**	**[45]**	**1**	**2.5**	**[45]**
Brier score	0	0		1	5.0	[45]	1	2.5	[45]
Nagelkerk's R^2	0	0		1	5.0	[45]	1	2.5	[45]

	Studies published up to 2011 (n=20)			Studies published after 2011 (n=20)			All Studies (N=40)		
Performance measures	n	%	Ref.	n	%	Ref.	n	%	Ref.
**Reclassification** [4]	**0**	**0.0**		**4**	**20.0**	**[45,46,59,62]**	**4**	**10.0**	**[45,46,59,62]**
Net reclassification improvement	0	0.0		4	20.0	[45,46,59,62]	4	10.0	[45,46,59,62]
Integrated discrimination index	0	0.0		2	10.0	[45,59]	2	5.0	[45,59]
**Clinically usefulness**	**3**	**15.0**	**[17,18,36]**	**8**	**40.0**	**[27,45,61,57,49,52,55,46]**	**11**	**27.5**	**[17,18,27,36,45,46,49,52,55,57, 61]**
Sensitivity/specificity	3	15.0	[17,18,36]	5	25.0	[27,45,55,57,61]	8	20.0	[17,18,27,36,45,55,57,61]
Decision curve	0	0.0		3	15.0	[49,52,46]	3	7.5	[49,52,46]
**No performance measure at all**	**11**	**55.0**	**[21,24-26,29, 30-33,35,40]**	**0**	**0.0**		**11**	**27.5**	**[21,24-26,29, 30-33,35,40]**

[1)] Two studies reported multiple calibration measures; [2)] Two studies reported multiple discrimination measures; [3)] One study reported both performance measures; [4)] Two studies reported both reclassification measures
